# Predictive value of C-X-C motif chemokine receptor 4-directed molecular imaging in patients with advanced adrenocortical carcinoma

**DOI:** 10.1007/s00259-024-06800-z

**Published:** 2024-06-19

**Authors:** Wiebke Schloetelburg, Philipp E. Hartrampf, Aleksander Kosmala, Sebastian E. Serfling, Niklas Dreher, Andreas Schirbel, Martin Fassnacht, Andreas K. Buck, Rudolf A. Werner, Stefanie Hahner

**Affiliations:** 1 Department of Nuclear Medicine, University Hospital Würzburg, Oberdürrbacher Str. 6, 97080 Würzburg, Germany; 2 Division of Endocrinology and Diabetes, Department of Medicine I, University Hospital, University of Würzburg, Wurzburg, Germany; 3 Division of Nuclear Medicine and Molecular Imaging, The Russell H. Morgan Department of Radiology and Radiological Science, The Johns Hopkins University School of Medicine, Baltimore, MD USA; 4 Department of Nuclear Medicine, Clinic for Radiology and Nuclear Medicine, Goethe University Frankfurt, University Hospital, Frankfurt, Germany

## Abstract

**Background:**

In patients affected with adrenocortical carcinoma (ACC), C-X-C motif chemokine receptor 4 (CXCR4) is highly expressed in sites of disease in an ex-vivo setting. We aimed to determine the predictive value of CXCR4-targeting [^68^Ga]Ga-PentixaFor PET/CT for outcome when compared to clinical parameters.

**Methods:**

We identified 41 metastasized ACC patients imaged with [^68^Ga]Ga-PentixaFor PET/CT. Scans were assessed visually and on a quantitative level by manually segmenting the tumor burden (providing tumor volume [TV], peak/mean/maximum standardized uptake values [SUV] and tumor chemokine receptor binding on the cell surface [TRB], defined as SUV_mean_ multiplied by tumor volume). Clinical parameters included sex, previous therapies, age, Weiss-Score, and Ki67 index. Following imaging, overall survival (OS) was recorded.

**Results:**

After [^68^Ga]Ga-PentixaFor PET/CT, median OS was 9 months (range, 1–96 months). On univariable analysis, only higher TRB (per 10 ml, HR 1.004, 95%CI: 1.0001–1.007, *P* = 0.005) and presence of CXCR4-positive peritoneal metastases (PM) were associated with shorter OS (HR 2.03, 95%CI: 1.03–4.02, *P* = 0.04). Presence of CXCR4-positive liver metastases (LM) trended towards significance (HR 1.85, 0.9–4.1, *P* = 0.11), while all other parameters failed to predict survival. On multivariable analysis, only TRB was an independent predictor for OS (HR 1.0, 95%CI: 1.00-1.001, *P* = 0.02). On Kaplan-Meier analysis, TRB above median (13.3 months vs. below median, 6.4 months) and presence of CXCR4-positive PM (6.4 months, vs. no PM, 11.4 months) were associated with shorter survival (*P* < 0.05, respectively). Presence of LM, however, was also linked to less favorable outcome (8.5 months vs. no LM, 18.1 months), without reaching significance (*P* = 0.07).

**Conclusions:**

In advanced ACC, elevated tumor chemokine receptor binding on the tumor cell surface detected through [^68^Ga]Ga-PentixaFor PET/CT is an independent predictor for OS, while other imaging and clinical parameters failed to provide relevant prognostic information.

## Introduction

Adrenocortical cancer (ACC) is a rare endocrine malignancy (incidence of 0.5 to 2 cases per million people per year) with poor overall prognosis (5y survival rate < 20% for ENSAT stage 4) [1, 2]. Radical surgery is the only option for cure, but even after complete resection up to 50% of the patients relapse locally or develop distant metastases [3]. As such, reliable prognostic biomarkers identifying patients prior to or under systemic treatment are intensively sought and those high-risks may be scheduled for change in oncological management or treatment intensification.

Among others, C-X-C motif chemokine receptor 4 (CXCR4) is highly expressed in patients affected with ACC and ex-vivo analyses provided evidence that the expression level on the tumor cell surface is tightly linked to the proliferation index [4]. In recent years, the diagnostic PET agent [^68^Ga]Ga-PentixaFor targeting this chemokine receptor has been extensively validated in haematological malignancies and solid tumors [5–7]. In this context, a feasibility study demonstrated significant CXCR4 expression in-vivo in ACC patients, providing complementary information to [^18^F]FDG PET regarding distant metastasis [8]. It remains elusive whether the chemokine receptor PET signal is also predictive for outcome.

As such, in the present study, we aimed to determine whether CXCR4-targeting [^68^Ga]Ga-PentixaFor PET/CT can identify patients with metastasized ACC at increased risk for shorter overall survival (OS), in particular when compared to other established clinical parameters.

## Materials and methods

### Patient population

We retrospectively searched our institutional PET/CT database and identified 41 patients with adrenocortical carcinoma imaged with [^68^Ga]Ga-PentixaFor, which were referred to our institution to identify candidates suitable for radioligand therapy (RLT) using [^177^Lu]Lu- or [^90^Y]Y-PentixaTher. Parts of this cohort have been published before to determine the diagnostic usefulness of [^68^Ga]Ga-PentixaFor PET/CT [6, 8, 9] but without investigating its predictive value. The study was performed in accordance with the Declaration of Helsinki and patients signed written informed consent before the examination. The local ethics committee waived the need for further approval due to the retrospective character of this study (no. 20,210,726 02). For this retrospective single-center investigation, we retrieved the following clinical items from our medical archive: sex, age at time of diagnosis, previous treatment lines, ENSAT-stage, Weiss-Score and Ki67 index [1, 10, 11]. Patients’ characteristics are summarized in Table 1.

**Table 1:** Patients’ characteristics. ^1^Weiss-Score is based on 9 histological criteria and a score ≥ 4 indicates adrenocortical cancer [27, 28]

Patient Characteristics	No. of Patients	% of All Patients	Median (range)
Number of patients	41		
Age at time of CXCR-imaging in years			49.2 (27–77)
Sex (F: M)	24 : 17	59% : 41%	
Location of ACC (R: L)	18 : 23	44% : 56%	
**ENSAT stage at initial diagnosis**			
I	2	5%	
II	8	24%	
III	14	34%	
IV	17	41%	
**Histopathological Scoring**			
Weiss-Score^1^ (*n* = 19)			8 (4–9)
Ki67 index (%; *n* = 39)			20 (3–80)
**Average of different treatments previous to CXCR4 imaging**		4.9	
Adrenalectomy	37	90%	
Radiation therapy	10	24%	
Mitotane	41	100%	
Chemotherapy	41	100%	
**OS** (after CXCR4-imaging) in months			9.0 (1–96)
**Metastases**	41	100%	
Lung	35	85%	
Liver	29	71%	
Lymphnode (local and distant)	26	63%	
Peritoneum	17	41%	
Bone	8	20%	
Soft tissue	3	7%	
Local recurrence	13	32%	

### Radiotracer synthesis

Following good manufacturing practice, [^68^Ga]Ga-PentixaFor was provided using a synthesis module (att Scintomics, Fürstenfeldbruck, Germany) and disposable single-use cassette kits (ABX, Radeberg, Germany), as described previously [12]. A Siemens Biograph mCT 64 or 128 (Siemens Healthineers, Erlangen, Germany) was used. Whole-body PET was performed 60 min after an injected activity of median 138 MBq (range, 90–156 MBq) [^68^Ga]Ga-PentixaFor and covered the area from the skull to the mid thighs. In the majority of the cases, we used a low-dose CT protocol for attenuation correction and anatomic coregistration (120 keV, 512 × 512 matrix, 3–5 mm slices, increment: 30 mm/s, pitch index: 0.8, and rotation time: 0.5 s). PET images were reconstructed including attenuation, random events, and scatter.

### Image interpretation

Experienced board-certified radiologist (WS) and nuclear medicine physician reviewed and analysed the images. A dedicated workstation and software package was used (syngo.via; V60A; Siemens Healthineers, Erlangen, Germany).

We performed a target lesion (TL) assessment by investigating the visually most intense TL. A maximum of three TL per organ system were analyzed. Three-dimensional volumes of interest (VOI) applying an isocontour threshold of 40% were manually placed on the TL, providing peak/mean/maximum standardized uptake values (SUV_peak_, SUV_mean_, SUV_max_) and tumor volume (TV in ml) [6, 13]. Tumor receptor binding on the cell surface (TRB) was calculated as follows:

1 $${\text{TRB}} = {\text{SU}}{{\text{V}}_{{\text{mean}}}}{\text{x TV}}$$

The bloodpool activity was measured by by placing a VOI in the aortic arch. A target-to-bloodpool-ratio (TBR) which was used to quantify the relative uptake of the tumor lesion compared to background activity was then calculated using the following equation [14]:

2 $$TBR = SU{V_{max(TL)}}/SU{V_{mean(Bloodpool)}}$$

### Statistical analysis

Statistical analysis was performed using GraphPad Prism (version 9.4.1, GraphPad Prism Software, La Jolla, CA). Descriptive results are displayed as mean ± SD in case of normal distribution or as median and range if data were not normally distributed. We applied uni- and multivariable Cox regression analyses including hazard ratios (HR), along with 95% confidence intervals to identify prognostic parameters. Kaplan-Meier survival curves were also calculated. *P* < 0.05 was considered statistically significant.

## Results

Key characteristics of patients are summarized in Table 1. Briefly, 41 patients (median age 49 years) were included and 24 (59%) of them were female. The initial ENSAT stage was IV in 17/41 patients (41%). Patient had undergone median 4.9 treatment lines prior to PET/CT, including surgeries, radiation therapies and chemotherapies. None of the patients had been treated with [^177^Lu]Lu- or [^90^Y]Y-PentixaTher before or after [^68^Ga]Ga-PentixaFor imaging. Median OS after [^68^Ga]Ga-PentixaFor PET/CT was 9 months (1–96 months) and 37/41 (90%) died within the observation period.

### Quantitative and visual findings

All 41 patients presented with metastases at time of scan: predominant tumor burden was located in the lung (35/41, 85%), followed by the liver (29/41, 71%), lymphnodes (26/41, 63%) and in the peritoneum (PM, 17/41, 41%). Quantitative assessment of [^68^Ga]Ga-PentixaFor PET-positive tumor lesions are shown in Table 2. The following values were derived: averaged SUV_max/peak/mean_ were 11.3 ± 5.8, 7.5 ± 3.6 and 6.5 ± 3.6, respectively. Mean TV was 116.2 ± 173.4, mean TRB was calculated with 744.4 ± 1087 and mean TBR was 6 ± 3.4.

**Table 2:** Quantitative PET parameters of tumor burden derived from [^68^Ga]Ga-PentixaFor. TV, tumor volume. TRB, Tumor receptor binding. TBR, target-to-bloodpool ratio. Presented as median and range in parentheses as well as mean and standard deviation (SD)

	Median (range)	Mean ± SD
SUV_peak_	6.2 (2.6–16.5)	7.5 ± 3.6
SUV_max_	9.3 (4.4–25.4)	11.3 ± 5.8
SUV_mean_	4.9 (2.5–17.6)	6.5 ± 3.6
TV	43.6 (1.9–817.2)	116.2 ± 173.4
TRB	433.9 (14.96–5370)	744.4 ± 1087
TBR	5.0 (2.4–13.7)	6.0 ± 3.4

### Quantitatively derived TRB and presence of peritoneal metastases are predictors for overall survival

**Fig. 1:**
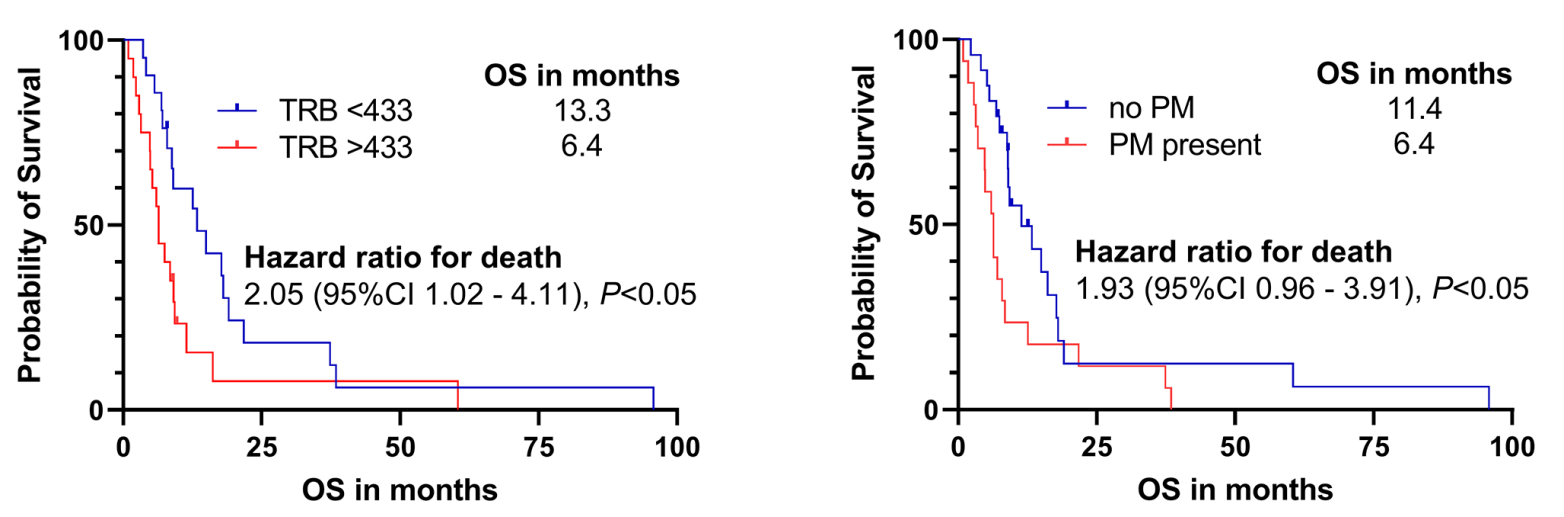
Kaplan-Meier plots for probability of overall survival (OS) using tumor receptor binding (TRB, left) and presence of peritoneal (PM, right) based on [^68^Ga]Ga-PentixaFor PET (right). Increased TRB was linked to shorter survival which was also observed for presence of CXCR4-positive PM. For TRB, median was used

**Fig. 2:**
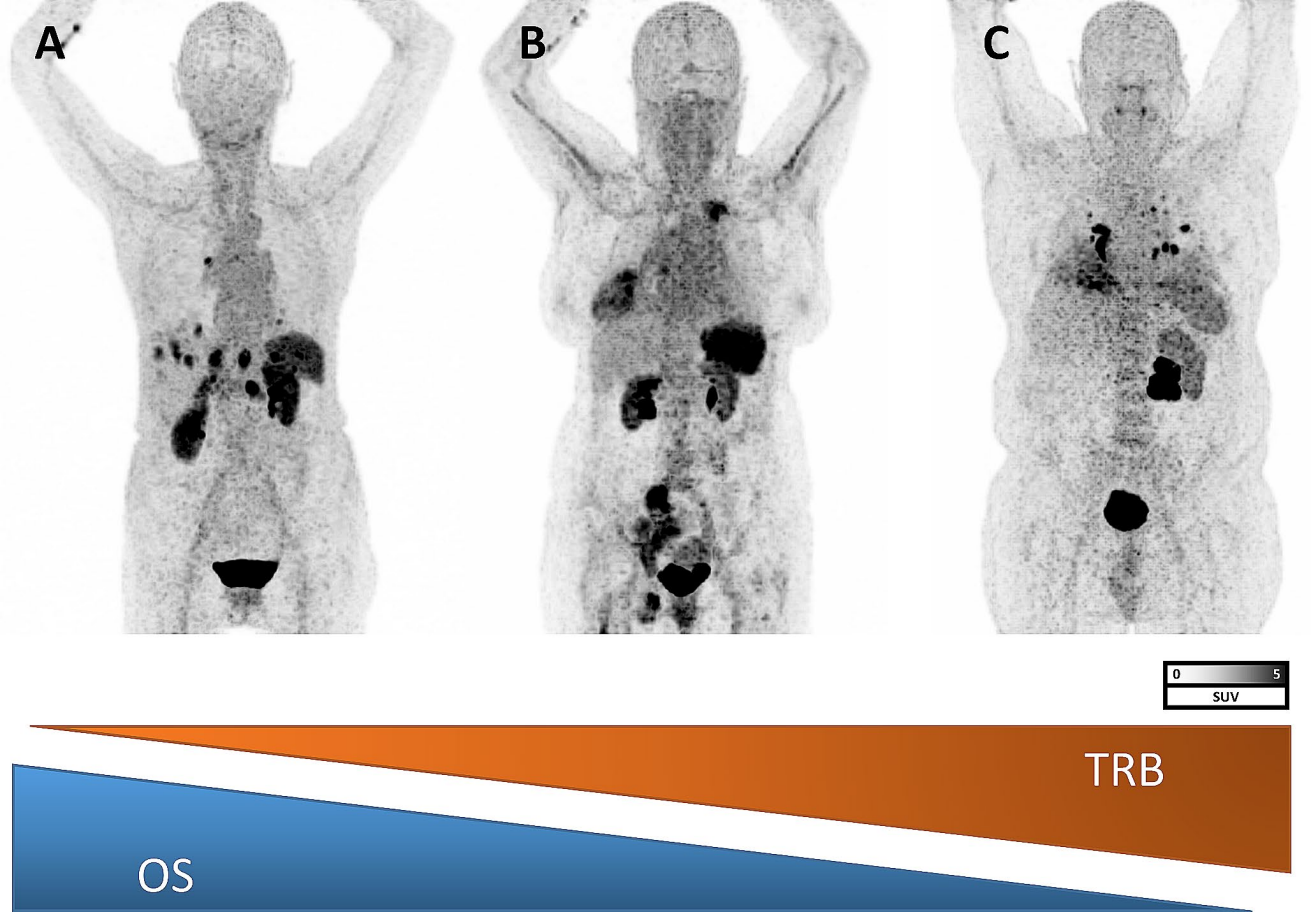
Example of three patients with [^68^Ga]Ga-PentixaFor PET/CT. Patient in **A** had a tumor receptor binding (TRB) of 259 and died 38 months after CXCR4-targeted molecular imaging, while Patient in **B** presented with TRB of 433 and survived 18 months. Patient **C** had the shortest overall survival (OS) with 9 months and the highest TRB of 645. Tumor volume, however, was virtually similar (A, 70 ml; B, 85 ml; C, 65 ml), thereby indicating that increased CXCR4 binding, but not PET-avid volume is of relevance for identifying high-risk patients

On univariable Cox regression analyses, only higher TRB (per 10 units, HR 1.004, 95%CI: 1.0001–1.007, *P* = 0.005) and presence of CXCR4-positive PM were significantly associated with shorter OS (HR 2.03, 95%CI: 1.03–4.02, *P* = 0.04). Presence of CXCR4-positive liver metastases trended towards significance (HR 1.85, 0.9–4.1, *P* = 0.11) while all other parameters failed to predict survival (*P* > 0.15). On multivariable Cox regression, only TRB was identified as significant independent prognostic factor for OS (HR 1.0; 1.00-1.001, *P* = 0.02), while presence of CXCR4-positive PM failed to reach significance (HR 1.9; 0.885–3.62, *P* = 0.09; Table 3). Kaplan-Meier analyses showed significant separation between patients with a TRB below or above a median of 433 (13.3 vs. 6.4 months, HR 2.05; *P* = 0.03) and for patients who presented with or without PM (11.4 vs. 6.4 months, HR 1.93; *P* = 0.04). Presence of LM, however, was also linked to less favorable outcome (8.5 months vs. no LM, 18.1 months), without reaching significance (*P* = 0.07) (Fig. 1 and 2).

**Table 3:** Univariable and multivariable Cox regressions (*HR* Hazard ratio, 95%*CI* confidence interval). Significant *P*-values are marked in bold. TRB, Tumor receptor binding. TBR, target-to-bloodpool ratio. TV, tumor volume

	Univariable			Multivariable		
	HR	95% CI	*P*-value	HR	95% CI	*P*-value
**TRB** (per 10 ml)	1.004	1.001–1.007	**0.005**	1.004	1.000–1.006	**0.02**
Peritoneal metastases	2.03	1.03–4.02	**0.04**	1.90	0.885–3.62	0.09
Liver metastases	1.85	0.90–4.10	0.11			
Local lymphnode metastases	1.61	0.83–3.26	0.15			
TBR	1.07	0.97–1.18	0.16			
Local relapse	1.62	0.78–3.24	0.18			
TV	1.00	1.00–1.003	0.22			
Bone metastases	1.53	0.64–3.20	0.31			
Weiss Score	1.17	0.83–1.70	0.39			
SUV_peak_	1.03	0.92–1.13	0.61			
SUV_max_	1.02	0.95–1.08	0.59			
Presence of primary tumor	0.83	0.35–1.80	0.64			
Lung metastases	0.83	0.37–2.23	0.68			
SUV_mean_	1.01	0.91–1.11	0.76			
ENSAT-stage	0.95	0.68–1.34	0.74			
Soft tissue metastases	1.18	0.28–3.33	0.79			
Distant lymphnode metastases	0.96	0.46–1.89	0.90			
Ki67 index	0.99	0.98–1.02	0.95			

## Discussion

In this study, including the largest cohort investigating [^68^Ga]Ga-PentixaFor in patients with metastatic ACC, an increased tumor receptor binding was independently associated with shorter survival. Notably, commonly established prognostic parameters such as Ki67 [1, 15] did not show prognostic relevance, demonstrating the potential of the chemokine receptor PET signal as a valuable prognostic marker in patients with advanced disease.

Due to proven upregulation in an ex-vivo setting, CXCR4-targeted [^68^Ga]Ga-PentixaFor PET/CT has entered the clinical arena for assessing sites of disease in patients with varying neoplasms, including hematological malignancies, solid tumors or benign pathologies [9, 16, 17]. For instance, a recent study demonstrated that [^68^Ga]Ga-PentixaFor PET exhibits the most intense uptake in advanced blood cancers, while among solid tumors, ACC revealed highest radiotracer accumulation [6]. Building on these encouraging findings, our aim was to determine whether the derived in-vivo PET signal could also serve as a predictive marker. The independent association of TRB, reflecting chemokine receptor density on the tumor cell surface, with a deteriorating outcome highlights the added value of molecular imaging targeting CXCR4 expression. This molecular imaging approach complements conventional imaging techniques like CT or MRI, which primarily focus on disease extent.

Relative to [^68^Ga]Ga-PentixaFor, established prognostic factors such as initial ENSAT stage, glucocorticoid excess or proliferation index had rather less prognostic relevance in our study [10, 18]. This finding may be partially explained by the fact that the vast majority of scans were conducted during treatment course, in particular to identify patients eligible for [^177^Lu]Lu- or [^90^Y]Y-PentixaTher. As such, our findings suggest that histopathological features derived at time of initial diagnosis have limited prognostic value at a later disease stage, e.g., due to selective pressures on tumor biology caused by previous treatment lines. In this regard, Ki67 derived from tumor specimen upon initial histological work-up also showed no association with survival in Cox regression analyses (Table 3). In contrast, a correlation of Ki67 with CXCR4 expression within the same tumor tissue was found in immunohistochemical analyses [4]. As such, an interim scan targeting CXCR4 on the tumor cell surface may serve as a prognostic biomarker after having initiated locoregional or systemic therapies.

Beyond its diagnostic potential, the increased uptake of [^68^Ga]Ga-PentixaFor in metastases of ACC may also pave the way for a theranostic approach using [^177^Lu]Lu/[^90^Y]Y-PentixaTher. Promising outcomes have been achieved in patients with multiple myeloma, T-cell or large B-cell lymphoma using this theranostic approach, including partial or complete remission in selected cases [19–21]. The increased uptake in ACC observed in this and previous studies may also trigger CXCR4-targeted RLT in this patient population [8]. However, this therapeutic option causes eradication of the stem cell niche and thus, stem cell backup is mandatory [22]. While such a bone marrow ablation may be an integral part of the treatment protocol in hematological malignancies [22], this phenomenon would be a major adverse event in ACC and would require harvesting stem cells preferably early in the treatment course, e.g., under first line chemotherapeutic protocols [23, 24].

Moreover, multiple pathways in ACC cells, such as glucocorticoid excess or WNT/ß-catenin upregulation are linked to immunoresistance [25–27], which may explain the limited therapeutic effect of PD-1/PD-L1 inhibitors. Of note, those pathways are also linked to CXCR4 expression suggesting that combined CXCR4-targeted therapies with immune checkpoint inhibitors may be of therapeutic interest. In such challenging clinical scenarios, [^68^Ga]Ga-PentixaFor may not only identify chemokine receptor binding in-vivo to determine individuals eligible for such a combination treatment, but may also provide prognostic potential in a manner similar to our findings in patients treated with varying systemic therapies.

Limitations of our investigation include the small number of patients and the retrospective character. Additionally, the diverse treatment lines prior to CXCR4-directed imaging pose a challenge. Nonetheless, ACC is a rare cancer [1], making data pooling of multiple study sites indispensable to further evaluate the clinical benefit of CXCR4-directed imaging in this patient population.

## Conclusions

In patients with advanced ACC, [^68^Ga]Ga-PentixaFor PET-based TRB reflecting chemokine receptor density on the tumor cell surface was independently associated with reduced overall survival. This chemokine receptor PET signal provides valuable prognostic information offering potential as a promising non-invasive tool for assessing disease progression and guiding treatment decisions in patients with metastatic ACC.

## Data Availability

Detailed information about the image analysis or the overall survivals of the subjects presented in this study are available on reasonable request from the corresponding author.
